# Bilateral Mandibular Simple Bone Cysts: A Report of an Unusual Case

**DOI:** 10.7759/cureus.107036

**Published:** 2026-04-14

**Authors:** Christina Zagkou, Konstantina-Eleni Alexiou, Konstantinos Katoumas, Nikolaos Kolomvos, Christos Angelopoulos

**Affiliations:** 1 Department of Radiology, National and Kapodistrian University of Athens School of Dentistry, Athens, GRC; 2 Department of Oral and Maxillofacial Surgery, National and Kapodistrian University of Athens School of Dentistry, Athens, GRC

**Keywords:** cbct, incidental findings, mandible, pseudocysts, simple bone cyst

## Abstract

Simple bone cysts are intraosseous cavities that are surrounded by bony walls, and they are either empty or contain liquid and/or connective tissue. They have similar radiological findings to those of true cysts, but they do not have an epithelial lining and are thus regarded as pseudocysts. They are frequently asymptomatic and most often an incidental finding on radiographic examination. The purpose of this case report is to present a rare case of bilateral occurrence of mandibular simple bone cysts.

A 23-year-old female patient presented to the Department of Oral Diagnosis and Radiology of the School of Dentistry, University of Athens. Her chief complaint was tenderness and discomfort in the posterior aspect of her mouth bilaterally. After taking medical history and conducting intraoral and extraoral examination, she was referred for panoramic radiography and cone beam computed tomography (CBCT). Upon examination of the panoramic radiograph, two large, well-defined, and well-corticated radiolucent lesions with scalloping along the roots of teeth were detected on the body of the mandible bilaterally. CBCT showed a slight buccal-lingual expansion of the cortical plates, as well as thinning of the lingual cortical plate. Bilateral simple bone cysts of the mandible are rare, and only a few have been reported in the literature. The prognosis is good, and recurrences are rare. Knowledge of clinical and radiographic features helps diagnose this entity successfully.

## Introduction

Simple bone cysts (SBCs) are intraosseous cavities encased by bony structures, which may either be devoid of contents or filled with fluid. They have similar radiological findings to those of true cysts, but they do not have an epithelial lining and are thus regarded as pseudocysts. They are frequently asymptomatic and most often an incidental finding on radiographic examination [1].

In 1946, Rushton established the first diagnostic criteria for simple bone cysts: a single lesion lacking an epithelial lining, no evidence of infection, and a cavity within bony walls that may contain fluid or soft tissue, without other pathological or chemical features that would exclude an SBC diagnosis [2]. These criteria are still widely accepted, though later reports have described cases with multiple lesions and cavities containing gas rather than fluid [3].

SBCs are rare. They have been reported more frequently in the long bones and, occasionally, in the jaws, comprising 1.25% of jaw cysts [4]. They mainly affect patients in their second decade of life, in men and women alike [5]. The etiology of these lesions is unclear. Despite the synonym “traumatic bone cyst,” trauma is not considered an etiological factor for simple bone cysts, as their occurrence in individuals with a history of trauma is comparable to that observed in the general population. Other hypotheses include developmental anomalies or a blockage of lymphatic drainage from venous sinusoids [6].

On radiographic examination, a simple bone cyst appears as a well-circumscribed radiolucent lesion, which may or may not show a sclerotic margin. SBCs are usually unilocular, and most are found in the mandible, especially in the posterior areas of its body, anywhere from the symphysis to the ramus. According to the literature, only 11% of SBCs present as multifocal lesions, with the first case reported by Hankey in 1947 [7]. In cone beam computed tomography (CBCT), slight bucco-lingual expansion of the cortical plates of the bone can be observed, as well as displacement of the inferior alveolar nerve canal. The most common radiographic finding of SBCs is the “scalloping effect,” where the cyst extends between the roots of the teeth [8]. Even though SBCs are usually an incidental radiological finding, there have been reports of swelling due to bone expansion (one fourth of the cases), pain (less than 10% of the cases), pathological fractures, and hypoesthesia of the inferior alveolar nerve [9].

Rarely, more than one cyst may be found in the same patient; a few cases have been described, usually associated with florid cemento-osseous dysplasia (FCOD) [10]. Although mechanistic links remain speculative, the association between these two entities remains unclear. In general, multiple jaw radiolucencies are more commonly linked to metabolic or endocrine disorders, such as hyperparathyroidism, or to multiple odontogenic keratocysts seen in Gorlin-Goltz syndrome. In this article, a case report of a bilateral occurrence of simple bone cysts with no association with cemento-osseous dysplasia or other metabolic disorders will be presented, contributing to the limited literature and underscoring the importance of careful diagnostic evaluation in such cases.

This article was presented as an e-poster at the 25th Congress of the International Association of Dental and Maxillofacial Radiology, June 25, 2025, London, UK. 

## Case presentation

A 23-year-old, white, female patient presented to the Department of Oral Diagnosis and Radiology at the University of Athens School of Dentistry (Athens, Greece) in March 2024. Her chief complaint was tenderness and discomfort around the back of her mouth bilaterally, present for the past two months, as well as a gradual reduction in mouth opening. The patient’s medical history was not contributory. On clinical examination, no palpable lymph nodes were detected, and intraoral and extraoral findings were within normal limits, apart from the reported symptoms. The patient was subsequently referred for panoramic radiography to assess the wisdom teeth.

**Figure 1:**
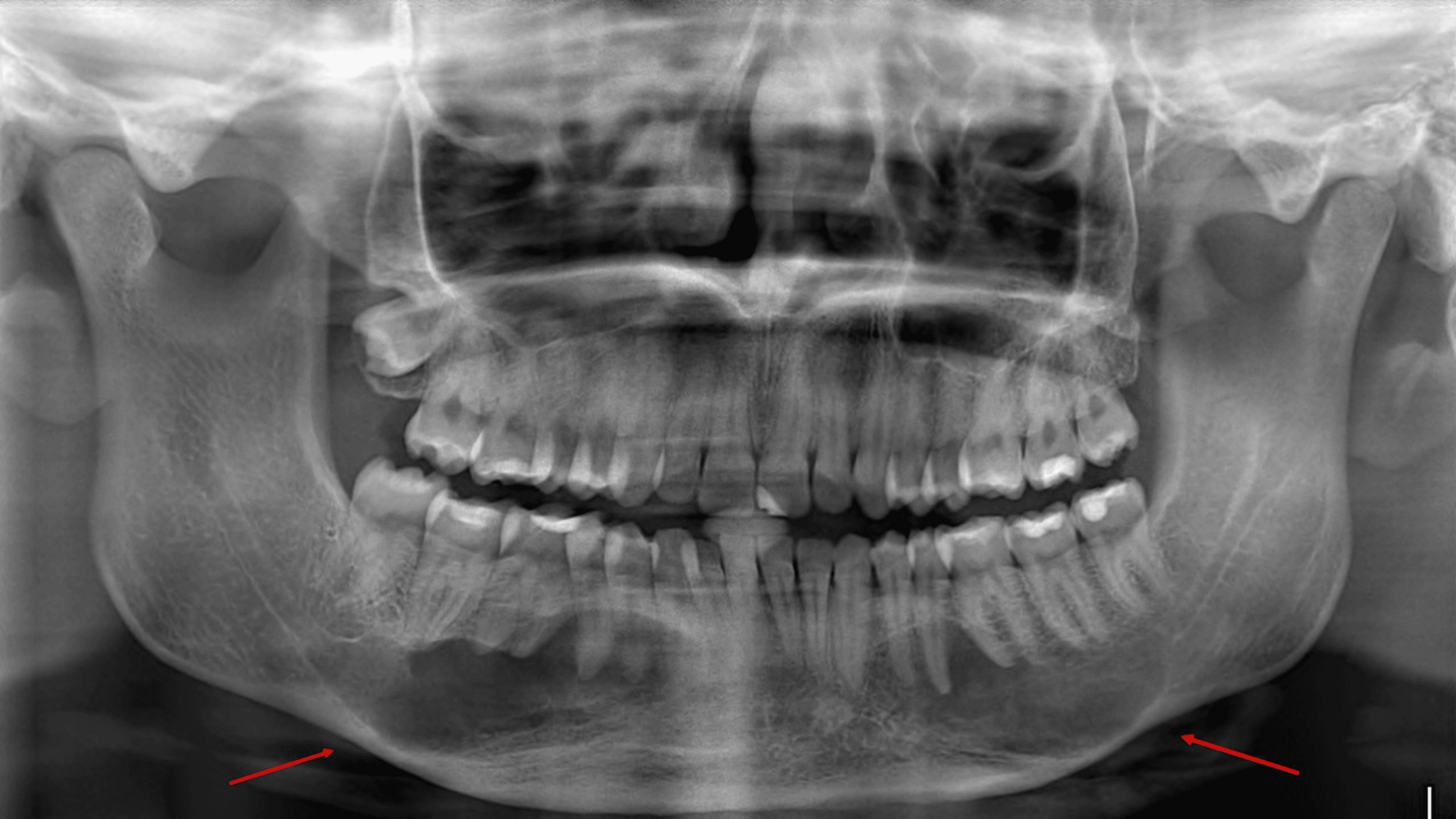
Preoperative panoramic radiograph, displaying two large radiolucent lesions with well-defined borders on the body of the mandible bilaterally (red arrows), which extend from the canines to the mesial root of the third molars, below their respective apexes

**Figure 2:**
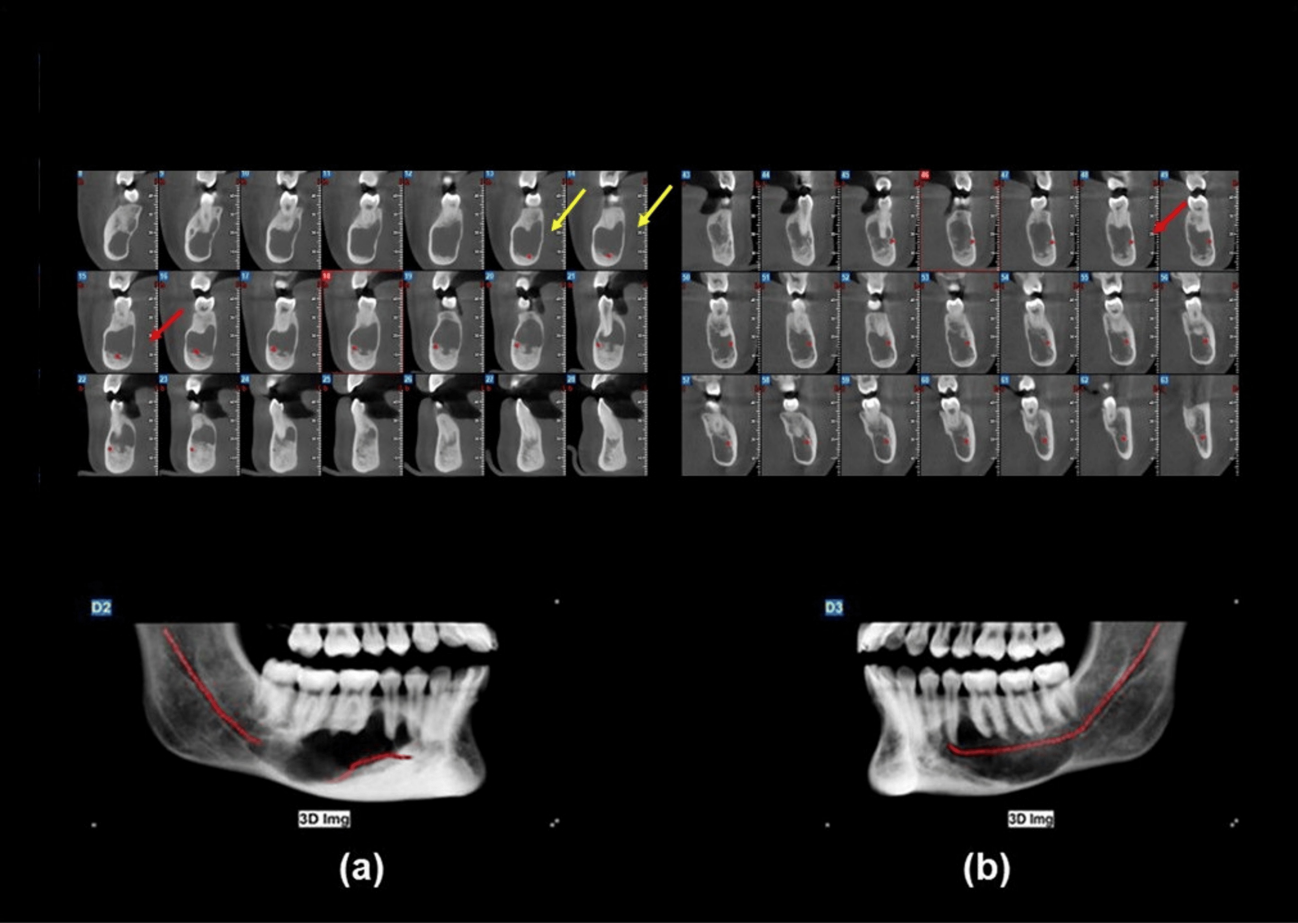
Cone beam computed tomography scan (CBCT) confirmed the presence of two well-corticated and well-defined radiolucent lesions, with a size of 44mm x 13mm bilaterally. A slight buccal-lingual expansion of the cortical plates was observed (red arrows), as well as thinning of the lingual cortical plate. The inferior alveolar nerve had also been displaced (yellow arrows), and no root resorption of the teeth was detected. (a) right side; (b) left side

Upon examination of the panoramic radiograph, two large radiolucent lesions with well-defined borders were discovered incidentally on the body of the mandible bilaterally, which extend from the canines to the mesial root of the third molars, below their respective apexes (Figure 1) She was then referred for a CBCT for further investigation, where the presence of the two well-corticated and well-defined radiolucent lesions was confirmed, with a size of 44mm x 13mm bilaterally. CBCT images were acquired with a Newtom VGi CBCT imaging unit (Cefla, Imola, Italy), using an 8cm x 8cm field of view for the maxilla and a 12cm x 8cm field of view for the mandible at 110kV, 2.69mA, with a 3.6s exposure time. A slight buccal-lingual expansion of the cortical plates was observed, as well as thinning of the lingual cortical plate. The inferior alveolar nerve had also been displaced from both sides, and no root resorption of the teeth was detected (Figure 2). The teeth in the region of the lesion were all vital, as confirmed by both cold testing and electric pulp testing.

The radiographic features were not specific for a single entity; therefore, the radiologic differential diagnosis included odontogenic keratocyst, SBC, central giant cell granuloma, and cementoma (periapical cemento-osseous dysplasia), first stage. Based on the exclusion of the aforementioned entities and the overall radiographic characteristics, a simple bone cyst was considered the most consistent diagnosis. Since third molar extraction had already been indicated due to clinical symptoms and impaction, the radiographic findings were regarded as incidental, and the patient was subsequently scheduled for the extraction of teeth #18, #28, #38, and #48 at the Department of Oral and Maxillofacial Surgery.

In April 2024, a posterior superior alveolar nerve block was performed for tooth #28, along with an inferior alveolar nerve block for tooth #38, using articaine hydrochloride 40 mg/mL and adrenaline 0.01 mg/mL, EP (Septanest 1.7 mL N50 - Septodont Co., Saint-Maur-des-Fossés, France). After successful extraction, a single 3-0 resorbable polyglactin 910 suture (VicrylTM, Ethicon Inc., Somerville, NJ, USA) was placed in the gingiva medial to the extracted maxillary and mandibular third molar sockets.

In the same surgical session, attention was then directed to the left intraosseous lesion, which had been identified radiographically. An incision was performed from the distal papilla of the left mandibular first molar around the cervical areas of the teeth to the mesial papilla of the left mandibular canine, with a vertical releasing incision at the mesial aspect of the canine. A two-sided subperiosteal flap was reflected to expose the underlying cortical bone, while respecting the mental nerve and foramen. A small cortical window was created in the lateral aspect of the alveolar bone, between the roots of the second premolar and first molar, using a sterile round bur No. 8 (Komet Dental, Gebr. Brasseler GmbH & Co. KG, Lemgo, Germany), under copious saline irrigation. Upon entry, the bony cavity was empty, with no fluid present, except for an exceedingly thin layer of connective tissue in certain areas. After curettage, small bone fragments and membrane portions were sent for microscopic analysis. Microscopy showed fibrous connective tissue without epithelial lining, consistent with a simple bone cyst. The operative findings also strongly indicated a diagnosis of simple bone cysts; consequently, no additional treatment was deemed appropriate beyond the curettage procedure. Following the formation of a stable blood clot, the mucoperiosteal flap was repositioned and secured with resorbable 4-0 polyglactin 910 sutures (Vicryl™, Ethicon Inc., Somerville, NJ, USA). The patient was then prescribed niflumic acid 250mg up to three times per day PRN, methylprednisolone 16 mg every 12 hours for 2 days, and amoxicillin/clavulanic acid (875/125) mg every 12 hours for 5 days. Finally, the patient was scheduled for regular follow-up to monitor healing and radiographic bone repair.

**Figure 3:**
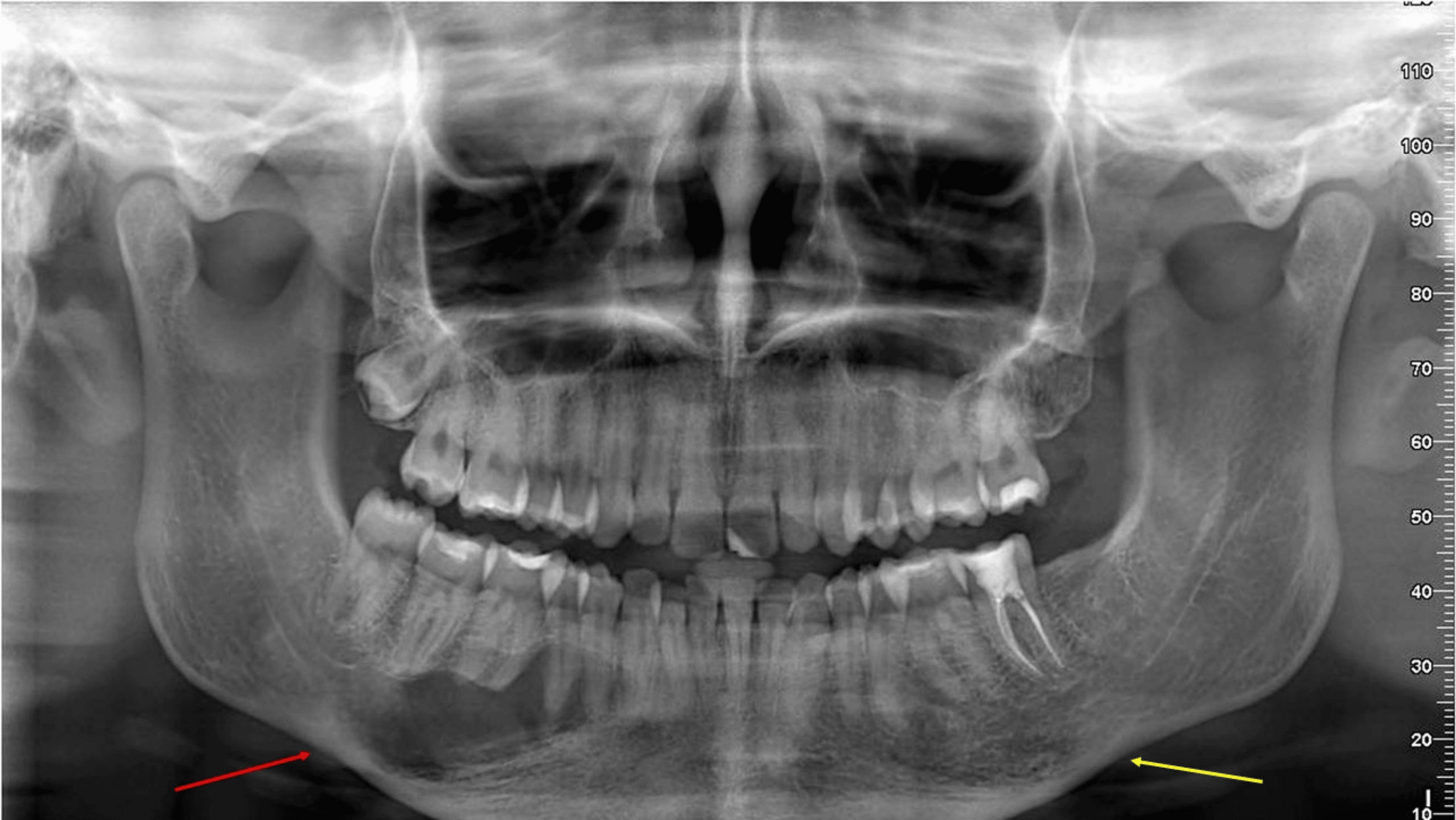
Postoperative radiograph, displaying a significant reduction in the size of the radiolucent lesion in the left body of the mandible (yellow arrow), indicating satisfactory bone healing and ongoing regeneration. In contrast, the radiolucent lesion in the right body of the mandible (red arrow) remains untreated, as surgical intervention was deferred at the patient’s request.

The patient experienced mild paresthesia of the left mental nerve that resolved over time. The post-surgical period was otherwise uneventful. Despite regular follow-up, the patient declined surgical intervention on the right side of the mandible and removal of the impacted third right maxillary molar, for the time being. One year postoperatively, a panoramic radiograph (Figure 3) revealed a significant reduction in the size of the radiolucent lesion in the left body of the mandible, indicating satisfactory bone healing. Progressive bone regeneration was evident, with restoration of regular trabecular pattern and cortical integrity. There was no alteration in the size of the radiolucent lesion on the right side of the mandible.

## Discussion

This case report presents radiographic characteristics that may pose a challenge in the definitive diagnosis of the lesions. The bilateral localization of the cyst could potentially steer the clinician’s mind further away from the diagnosis of SBCs, given their rarity, accounting for only 0-1.25% of all jaw cysts, and their typically unilocular presentation [11]. Based on these figures, multiple SBCs represent approximately 0.14% of all jaw cysts, underscoring their exceptional rarity. Therefore, a definitive diagnosis requires careful clinical, radiological, and histological evaluation. In this case report, the differential diagnosis should primarily include entities known to present as multiple or bilateral radiolucencies of the jaws. Odontogenic keratocysts may present as multiple lesions, most commonly in association with syndromes such as Gorlin-Goltz syndrome, with only 5% of them being non-syndromic [12]. They are characterized by potentially aggressive behavior, with a tendency for significant bone expansion and possible involvement of adjacent teeth, including root resorption. Thus, the absence of root resorption and lack of any syndromic association in the patient’s history made the diagnosis of an odontogenic keratocyst less likely. Early-stage cemento-osseous dysplasia was considered due to the radiolucent appearance and the vitality of the associated teeth. However, this entity typically presents as small, well-defined lesions confined to the periapical region, most commonly in the anterior mandible of middle-aged women. In the present case, the lesions appeared significantly more extensive, rendering this diagnosis less likely. Central giant cell granulomas are rare, benign but locally aggressive lesions that may occasionally present bilaterally, typically affecting children and young adults. They most commonly involve the anterior mandible, often crossing the midline, and are frequently associated with clinical features such as pain, rapid growth, tooth displacement, and root resorption [13]. In the present case, the absence of clinical signs, such as swelling, the lack of aggressive radiographic features, such as root resorption, and the posterior distribution of the lesions made the diagnosis of central giant cell granuloma also unlikely. It should be noted that small, incidentally discovered traumatic bone cysts may be radiographically indistinguishable from early lesions of other types [14].

In 2014, Seo-Young et al. reviewed the literature on multiple SBCs in the jaws and noted that by that year, only 34 cases had been reported [15]. The parameters they examined included the patient’s age, sex, symptoms, history of trauma, vitality of the involved teeth, cavity contents, histology, treatment outcomes, number and location of lesions, and their radiographic appearance. Echoing Seo-Young et al.’s findings, our case also involves localization on the posterior mandibular body, female gender, and young age, characteristics shared by both multiple SBCs and unilocular lesions. The study revealed that these characteristics were consistently observed in all examined cases of multiple SBCs. Diverging from prior reports where almost half of the patients reportedly presented with fluid at aspiration, in our case, the cavity was completely empty.

Furthermore, in our case, no history of trauma was present, and the same applied to all those 34 cases. This could be interpreted by taking into consideration the fact that trauma rarely takes place bilaterally. The authors also suggested a possible underlying systemic predisposition to vascular abnormalities in patients with multiple SBCs; however, this hypothesis was not supported in our case, as no relevant findings were identified in the patient’s medical history. Within the same cohort, a significant difference was observed in the reported frequency of bone expansion, which was noted in fewer than half of the patients and was typically associated with multilocular radiographic appearances of the lesions. Prior to 2012, diagnoses of multiple SBCs were largely based on 2D imaging, such as panoramic and periapical radiographs, which have limited accuracy in evaluating bone expansion. This likely led to an underestimation of the frequency and extent of cortical involvement. In this case, CBCT revealed minor buccolingual expansion of the cortical plates and thinning of the lingual cortical plate. The use of CBCT in this case provided additional diagnostic information compared to conventional two-dimensional imaging, particularly in the assessment of subtle buccolingual cortical changes and cortical thinning that may not be fully appreciated on panoramic radiographs. This enhanced imaging detail contributed to a more accurate evaluation of the extent of the lesions and supported surgical planning. The lesions, however, did not exhibit the previously mentioned multilocular radiographic presentation, suggesting that these findings are representative of a minority of cases.

A more recent report published after 2014 further supports these observations, confirming that multiple simple bone cysts remain a rare presentation, most commonly affecting young patients and involving the posterior mandible [16]. These cases are frequently asymptomatic and discovered incidentally, with clinical and radiographic features largely overlapping those of solitary lesions. Although bilateral involvement continues to be uncommon, the available literature reinforces the variability in presentation, particularly regarding cavity contents and the presence or absence of cortical expansion. The present case is consistent with these findings and contributes to the limited number of reported bilateral cases without associated cemento-osseous dysplasia. 

Curettage of the bone walls is widely accepted as the gold standard for the management of SBCs of the jaws, as it aims to induce blood clot formation and facilitate subsequent bone regeneration. Additionally, alternative treatments, such as methylprednisolone acetate, application of Gelfoam (Pfizer, NY, USA), allogenic bone grafting combined with platelet-rich plasma, and various intralesional injection techniques, have been reported to yield favorable outcomes, in selected cases, particularly for smaller or asymptomatic lesions [17].

Recurrences are generally rare. According to Suei et al., most recurrences occurred within five months of surgical treatment, and this group recommended that follow-up continue until radiographic healing was confirmed within three years [18]. Importantly, in the majority of reported cases, including long-term follow-up studies, progressive trabecular bone regeneration is observed following surgical intervention, with gradual restoration of normal bone architecture over time. It has also been reported that recurrences were more common in cases of multiple SBCs than in solitary cases [19]. In 1986, Campanacci et al. established that the frequency of recurrences was threefold higher in multilocular lesions than in unilocular lesions, and it was also twice as prevalent when the radiographic measurement exceeded 21 cm. Finally, large lesions exhibiting scalloping, such as the one described in this case, can interfere with the formation of a stable blood clot required for bone healing, which may account for their persistence or tendency to recur [20].

## Conclusions

In conclusion, this case highlights a rare presentation of bilateral simple bone cysts of the mandible in the absence of associated cemento-osseous dysplasia, contributing to the limited literature on such occurrences. Given that simple bone cysts typically present as unilateral lesions, this manifestation underscores the importance of including them in the differential diagnosis of multiple radiolucent jaw lesions, which may otherwise mimic pathologies requiring more invasive management. Accurate diagnosis relies on the careful correlation of clinical, radiographic, and surgical findings, with CBCT providing valuable additional information for assessing lesion extent and subtle cortical changes. Furthermore, this case supports the effectiveness of conservative surgical exploration and curettage, while emphasizing that awareness of such atypical presentations helps clinicians avoid misdiagnosis and unnecessary overtreatment. Although a one-year follow-up demonstrated satisfactory bone healing in the treated area, longer-term radiographic surveillance is recommended in line with published literature. Overall, the present work expands the known spectrum of simple bone cysts and reinforces the necessity of a systematic diagnostic approach in multifocal cases.
